# Supplementary material for: Protocol for assessment of the efficiency of CRISPR/Cas RNP delivery to different types of target cells
Source: PLoS One. 2021 Nov 9;16(11):e0259812. doi: 10.1371/journal.pone.0259812 (PMC8577758; doi:10.1371/journal.pone.0259812)
Supplement: Attachment — Submitted filename: Response to Reviewers.docx [file pone.0259812.s001.docx]

Response to Reviewer #1 comments:

Dear Reviewer,

We want to thank you for the thoughtful feedback. We have incorporated all of your comments to our revision, tried to eliminate most of repeated sections and added some points to discussion. We also provide a point-by-point reply to all of your comments.

***Comment 1:***

We’ve carefully revised the repeated sections and put them together.

***Comment 2:***

We’ve explained the meaning of the last column and row in Table 4-6.

We suggest that there is no need to elevate concentration of glycerol above 10%, glycine – above 135 mM, betaine – above 270 mM and proline – above 180 mM, as these values exceed the optimal ones 1.8-45 times. Whether it is possible to increase the concentration of the reagents to such a high level or not we will see when we will be performing viability tests. The main aim is to optimize delivery conditions and we need to evaluate boundary (extreme) parameters.

***Comment 3:***

More discussion about potential efficiency, viability, large-scale production under different conditions including transfection reagents, electroporation, and so on was added.

***Comment 4:***

If the final selected protocol could not meet all the criteria mentioned above, the scalable one with the best indexes in protein delivery and genome editing efficacy, post-transduction recovery should be preferably considered for further usage. We’ve also added this phrase to discussion.

***Comment 5:***

All similar protocols were put together.

***Comment 6:***

Protocols for CD4/CD8 cells and CD34 cells also were put together. The main goal of our future study is optimization of CRISPR/Cas delivery, and we hope this will help us to design a specific protocols for CD4/CD8 cells and CD34.

On behalf of authors, I want to thank Reviewer #1. All comments were very helpful.

Best regards,

Marina Tyumentseva.

Response to Reviewer #2 comments:

Dear Reviewer,

On behalf of all co-authors, I thank you for your time and efforts on revising the manuscript. We now submit the revised text. We’ve carefully revised our manuscript according to your suggestions.

***Comment 1:***

In the introduction, we’ve described current methods used for CRISPR/Cas delivery by citing relevant literatures. Our innovation is that we will perform the first extensive comparative study of popular current methods and protocols of CRISPR/Cas RNP delivery to human cell lines and primary cells. Also, we will perform optimization of these methods and will try to offer new approaches in CRISPR/Cas RNP delivery (e.g., RBCs ‘ghosts’). Optimized and intermediate methods will be systematically characterized (delivery efficacy, editing efficacy, post-delivery viability, CRISPR/Cas RNP half-life and toxicity, etc.).

***Comment 2:***

We’ve tried to add more details to methods mentioned in our manuscript. Electrical transfer conditions were indicated for Neon Transfection System, while for 4D-Nucleofector system we’ve indicated only programs (as 4D-Nucleofector is a closed system). Also, we’ve added more description to cytotoxicity, viability, delivery and genome-editing efficacy tests. For each delivery procedure the amount of CRISPR/Cas RNP was added.

***Comment 3:***

We completely agree that editing efficiency is an important criterion. Editing efficiency was added to Fig 1 and text along side with delivery efficiency and other criteria.

***Comment 4:***

We’ve added chapters “4.6. Scalability assessment”, “4.7. Cost-effectiveness assessment” and “4.8. IP rights analysis” regarding evaluation of such criteria as scalability of delivery process, cost-effectiveness of the delivery process and intellectual property rights to our manuscript.

We want to thank Reviewer #2 as all suggestions helped us to revise our manuscript.

Best regards,

Marina Tyumentseva.
